# Combinatory Evaluation of Transcriptome and Metabolome Profiles of Low Temperature-induced Resistant Ascites Syndrome in Broiler Chickens

**DOI:** 10.1038/s41598-017-02492-8

**Published:** 2017-05-24

**Authors:** Shourong Shi, Yiru Shen, Shan Zhang, Zhenhua Zhao, Zhuocheng Hou, Huaijun Zhou, Jianmin Zou, Yuming Guo

**Affiliations:** 1 Poultry Institute, Chinese Academy of Agricultural Sciences, Yangzhou, Jiangsu 225125 China; 2 State Key Laboratory of Animal Nutrition, College of Animal Science and Technology, China Agricultural University, Beijing, 100093 China; 3 Department of Animal Science, University of California, Davis, CA 95616 USA; 4 Jiangsu Co-innovation Center for Prevention and Control of Important Animal Infectious Diseases and Zoonoses, Yangzhou, Jiangsu 225009 China

## Abstract

To select metabolic biomarkers and differentially expressed genes (DEGs) associated with resistant-ascites syndrome (resistant-AS), we used innovative techniques such as metabolomics and transcriptomics to comparatively examine resistant-AS chickens and AS controls. Metabolomic evaluation of chicken serum using ultra-performance liquid chromatography-quadruple time-of-flight high-sensitivity mass spectrometry (UPLC-QTOF/HSMS) showed significantly altered lysoPC(18:1), PE(18:3/16:0), PC(20:1/18:3), DG(24:1/22:6/0:0), PS(18:2/18:0), PI(16:0/16:0), PS(18:0/18:1), PS(14:1/14:0), dihydroxyacetone, ursodeoxycholic acid, tryptophan, L-valine, cycloserine, hypoxanthine, and 4-O-Methylmelleolide concentrations on day 21 and LysoPC(18:0), LysoPE(20:1/0:0), LysoPC(16:0), LysoPE(16:0/0:0), hypoxanthine, dihydroxyacetone, 4-O-Methylmelleolide, LysoPC(18:2), and PC(14:1/22:1) concentrations on day 35, between the susceptible and resistant groups. Compared to the susceptible group, transcriptomic analysis of liver samples using RNA-seq revealed 413 DEGs on day 21 and 214 DEGs on day 35 in the resistant group. Additional evaluations using gene ontology (GO) indicate that significant enrichment occurred in the oxygen transportation, defensive reactions, and protein modifications of the decreased DEGs as well as in the cell morphological formation, neural development, and transforming growth factor (TGF)-beta signalling of the increased DEGs on day 21. Oxygen transportation was also significantly enriched for downregulated DEGs on day 35. The combinatory evaluation of the metabolome and the transcriptome suggests the possible involvement of glycerophospholipid metabolism in the development of resistant-AS in broilers.

## Introduction

Ascites syndrome (AS) is a metabolic condition occurring in chickens, and its incidence has escalated globally over the past several years. Meat-type chickens are susceptible developing to AS because the desire for quick growth encourages the genetic selection of an intensive nature, exposure to extreme climatic conditions including low temperatures and elevated altitudes or fortified nutrition regimens with high-calorific diets^1^. Previous studies have indicated ascites-related mortality rates of 5% and 20% in broilers and roaster birds, respectively, resulting in serious economic losses^2^.

Recent studies have reported that AS-related animal deaths can be attributed to increased stress on the metabolic system. The rearing practices impose a considerable strain on chickens targeted as meat sources during their accelerated growth, leading to high tissue demand for oxygen, leading to subsequent hypoxic conditions and ultimate reduction of saturated blood oxygen with elevated haematocrit values^3, 4^. Therefore, it is necessary to develop broilers that are resistant to AS (resistant-AS) in the industry.

In the last few years, emerging “omics” technologies including metabolomics, proteomics, and transcriptomics, have shown considerable potential for identifying changes in biochemistry and signal transduction mechanisms associated with the development of diseases. Metabolomic profiling is an efficient strategy for determining the effects of cell perturbations on products of metabolism by analysing the differences in their concentrations^5^. In addition, transcriptomic profiling concurrently evaluates thousands of genes to generate a comprehensive analysis of the effect of exogenous elements on gene expression^6, 7^. Consequently, combining metabolomic and transcriptomic profiling is a key method contributing to improving the current knowledge of the mechanisms of intricate biological processes^8, 9^. Furthermore, these strategies have facilitated the effective investigation of different diseases^8, 9^ and ecological toxins^10, 11^, as well as nutritional interventions^12, 13^.

To identify metabolic biomarkers and DEGs related to resistant-AS, we established a chicken AS model and resistant-AS model, by exposing them to low temperatures, which were subsequently analysed the serum metabolome panel by applying an ultra-performance liquid chromatography-quadruple time-of-flight high-sensitivity mass spectrometry (UPLC-QTOF/HSMS) method. Furthermore, we used RNA sequencing (RNA-seq) to elucidate the transcriptomic liver panel and examined the consequences using biochemical and histological methods.

## Results

### Establishment of AS and resistant-AS models

**Figure 1:**
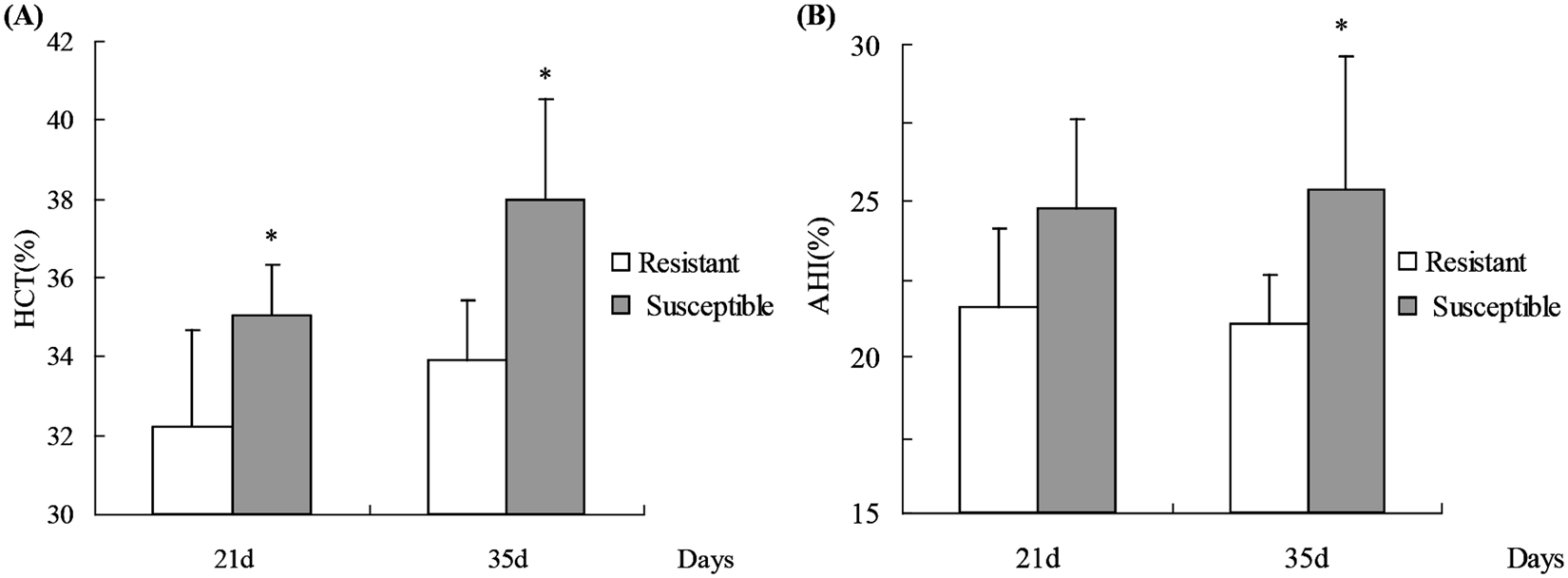
Haematocrit (HCT) and ascites heart index (AHI) of resistant and susceptible groups. (**A**) HCT and (**B**) AHI. Data are mean ± standard deviation (SD). *P < 0.05.

Figure 1 shows the hematocrit (HCT) and ascites heart index (AHI) values in the susceptible group and the resistant group on days 21 and 35. Compared to the susceptible group, the HCT values were significantly decreased in the resistant group on days 21 and 35 (Fig. 1A, P < 0.05), and the AHI value was significantly decreased on day 35 (Fig. 1B, P < 0.05), whereas the body weights on days 21 and 35 did not differ significantly (P > 0.05) between the two groups.

**Figure 2:**
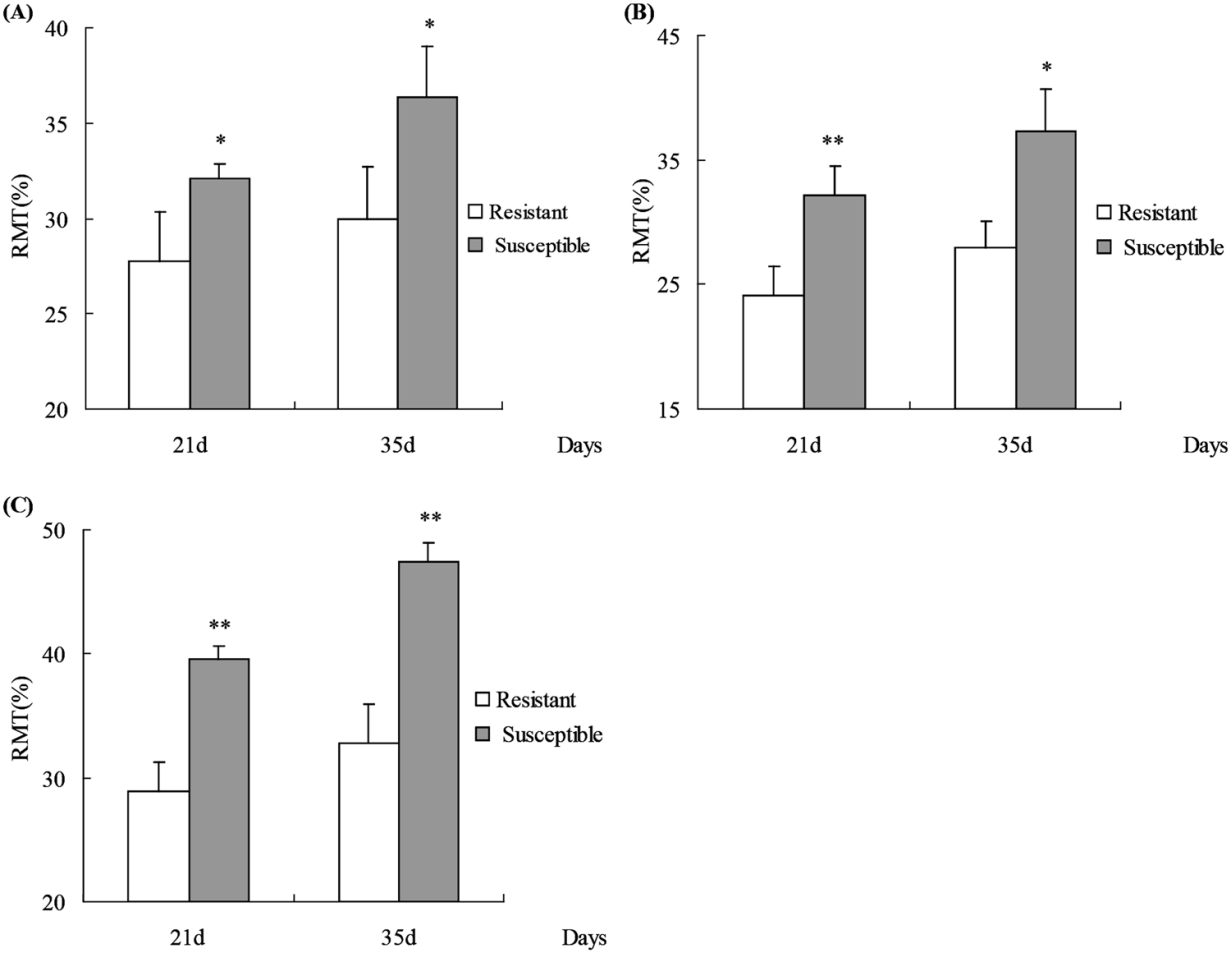
Relative medial thicknesses (RMT) of pulmonary arterioles of resistant and susceptible groups. (**A**) 100–200 μm; (**B**) 50–100 μm; (**C**) 20–50 μm. Data are mean ± standard deviation (SD). **P < 0.01 and *P < 0.05.

**Figure 3:**
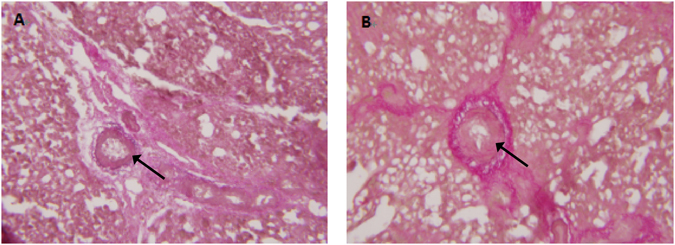
Histological differences in structure of pulmonary arterioles with external diameters of 20–50 μm (Weigert-Van Gieson ×100), between (**A**) resistant and (**B**) susceptible birds. Magnification, ×200.

Figure 2 shows the change in the relative medial thickness (RMT) of pulmonary artery samples from the susceptible and resistant chicken models on day 21 and 35, which was significantly thinner in the resistant chickens (external diameter, 100–200 μm) than it was in the susceptible chickens on day 21 and 35 (P < 0.05, Fig. 2A). As shown in Fig. 2B, the RMT of the lung artery (external diameter, 50–100 μm) was extremely significantly thinner on day 21 (P < 0.01) and significantly thinner on day 35 (P < 0.05) in the resistant group than it was in the susceptible group. As shown in Fig. 2C, the RMT of the lung artery (external diameter, 20–50 μm) was extremely significantly thinner on day 21 and 35 (P < 0.01). Figure 3 shows the comparison between the morphological variability of the lung arterioles, with outer diameters of 20–50 μm, of the resistant and susceptible chicken models. Compared to the susceptible group, the RMT of the pulmonary artery (external diameter, 20–50 μm) in the resistant group was significantly thinner, and the luminal change was not significant.

### Metabolomic Alterations Induced by AS and resistant-AS

**Figure 4:**
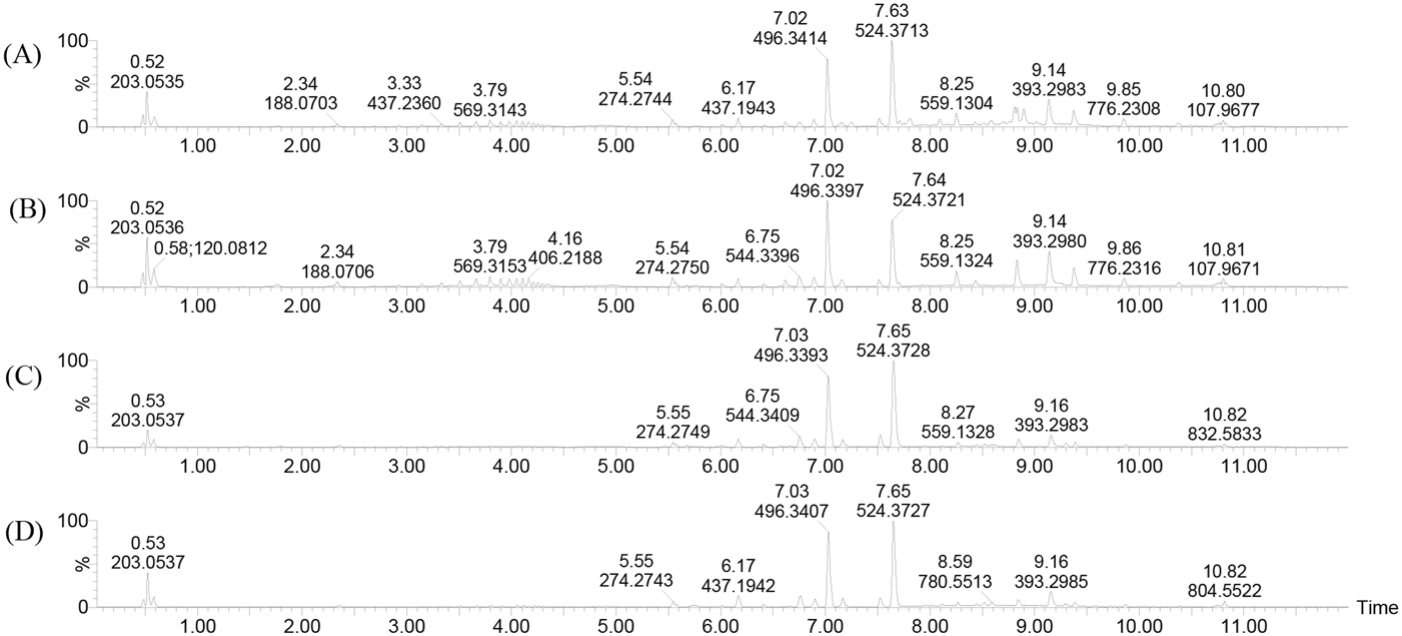
Positive ion base peak intensity (BPI) chromatogram of representative serum samples from (**A**) resistant and (**B**) susceptible groups sampled on day 21, and (**C**) resistant and (**D**) susceptible groups sampled on day 35.

**Figure 5:**
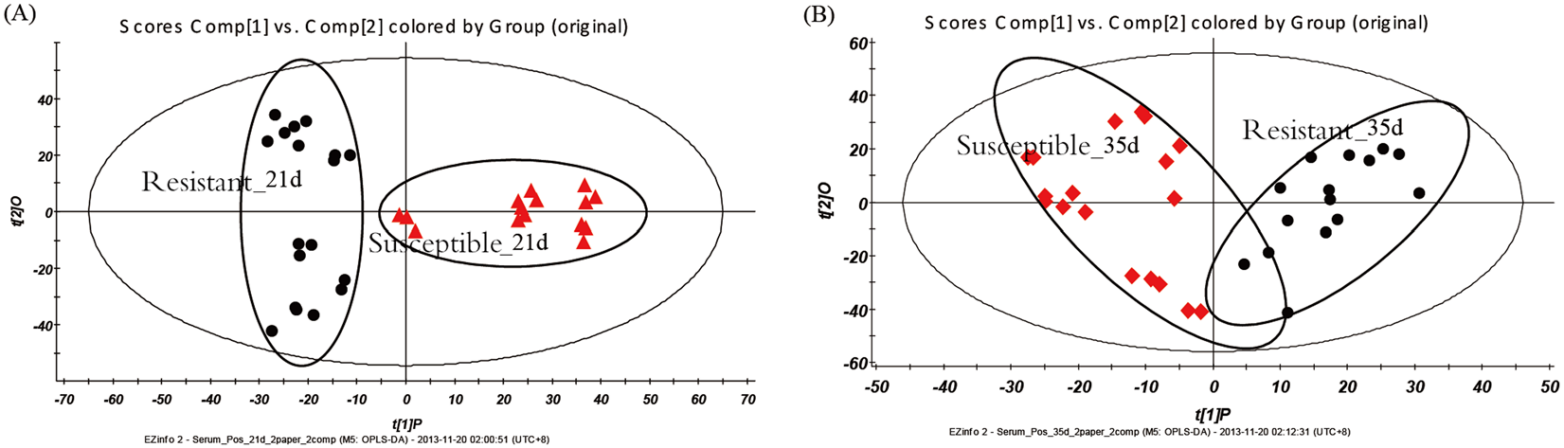
OPLS-DA plots using the scores from the first two principal components, (**A**) exhibiting the scatter between the resistant group and susceptible group on day 21; (**B**) exhibiting the scatter between the resistant group and susceptible group on day 35.

**Figure 6:**
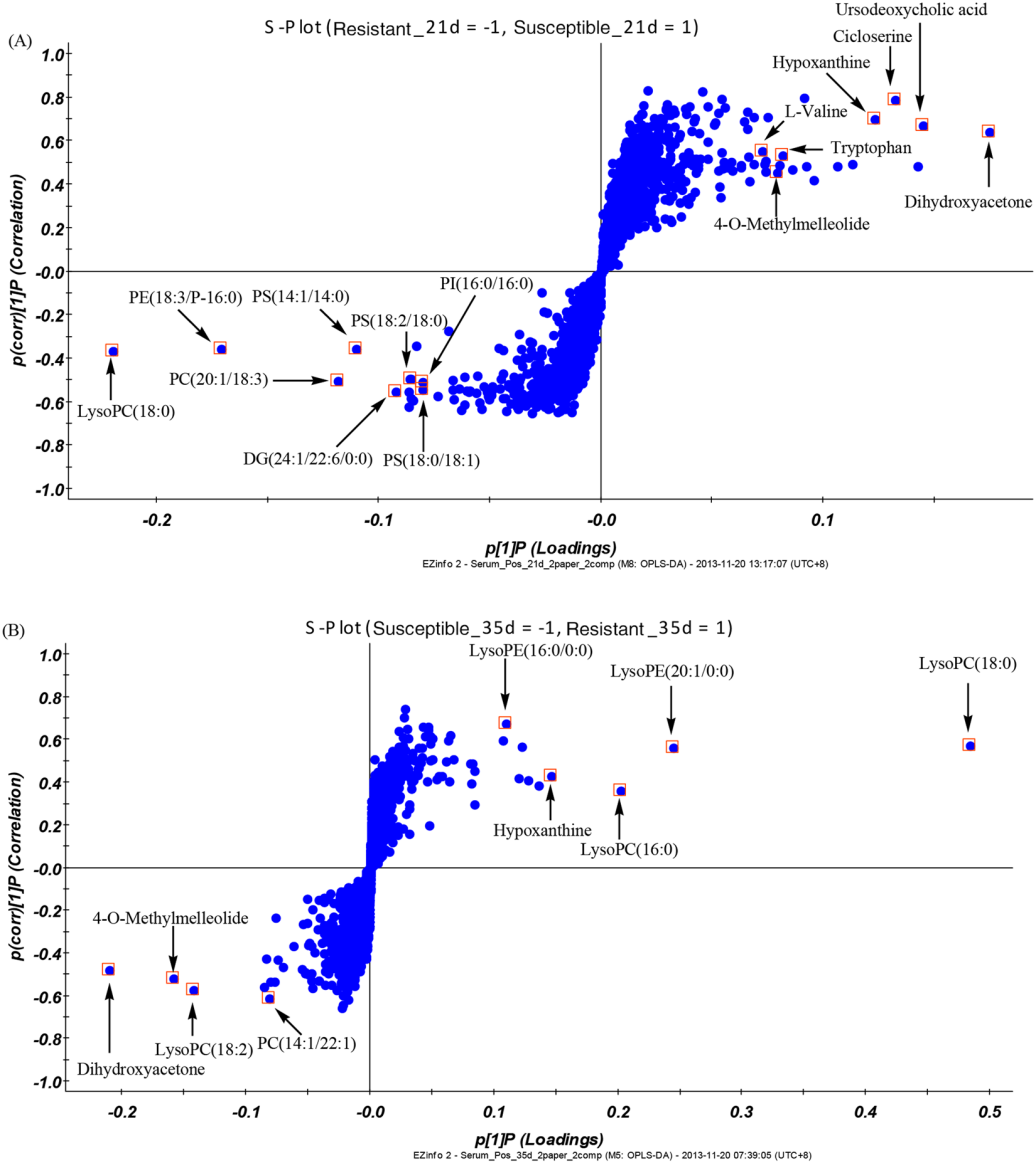
S-plot used in biomarkers selection on day (**A**) 21 and (**B**) 35. Variables marked (□) are metabolites selected as potential biomarkers. Variables far from the origin contributed significantly to differentiate the clustering of susceptible group from that of resistant group, and were considered potential biomarkers. Numbers consistent with Tables 1 and 2.

**Figure 7:**
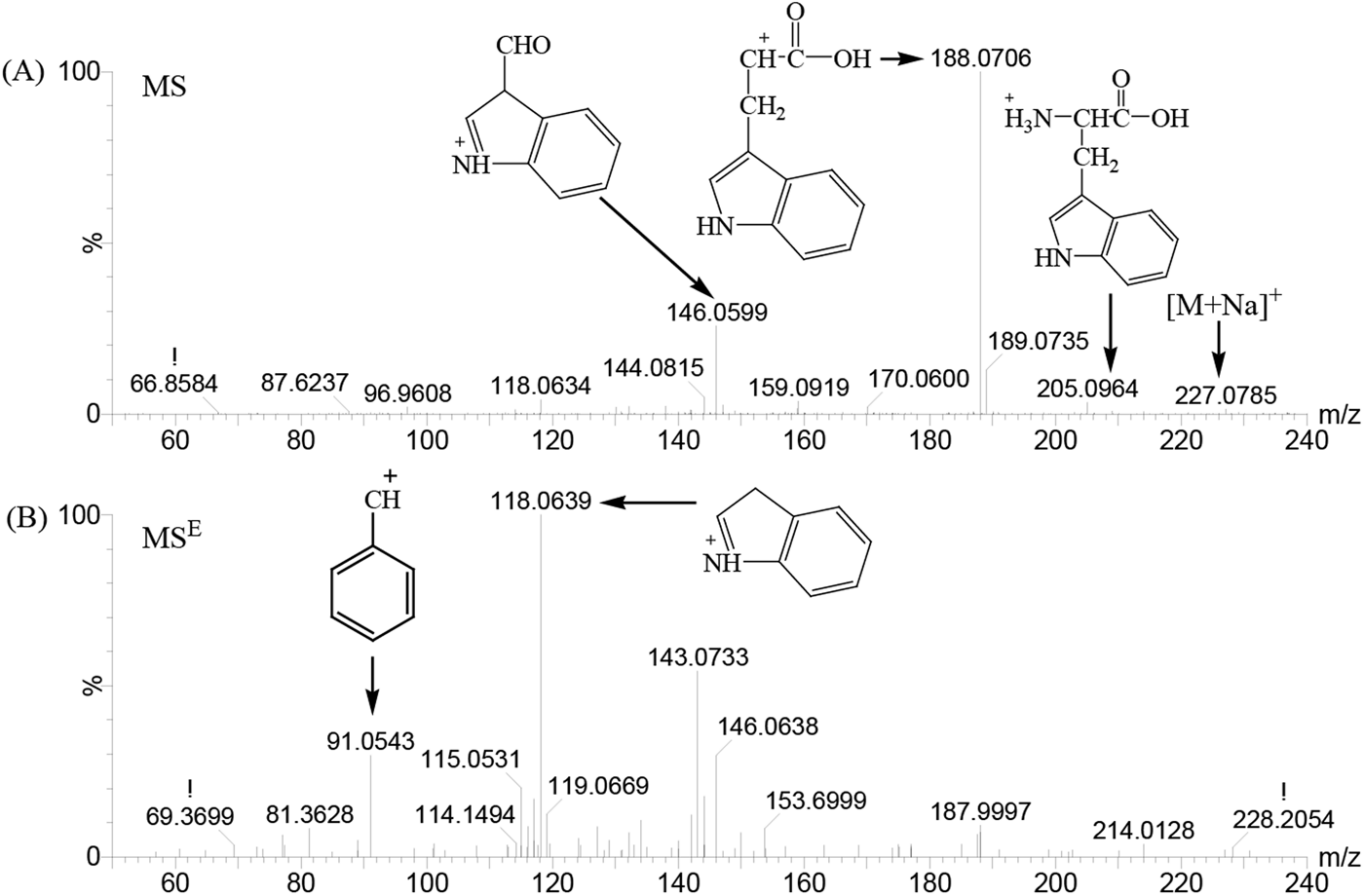
MS (**A**) and MS^E^ (**B**) of biomarker- tryptophan at m/z 188.0706 in the positive ion mode.

Figure 4 shows selected chromatograms of the serum positive base peak intensity (BPI) of the resistant and susceptible models on day 21 and 35. As shown in Fig. 4, the chromatographic peaks of all components in the sample were clear, and the separated effect was preferable. The results of the serum sample measurements were further evaluated using an orthogonal partial least squares discriminant analysis (OPLS-DA) using UPLC Q-TOF/MS, which identifies discriminatory variables based on the OPLS weight analysis. Figure 5 illustrates the positive ion mode score and loading plots constructed from measurements of serum from the resistant and susceptible chicken models on day 21 and 35. The score plot reveals that the resistant and susceptible chickens exhibited markedly different results, suggesting that there was a significant difference in the serum composition of the susceptible and resistant groups. The S loading plot enables biomarkers to be chosen visually. Figure 6 illustrates the respective S-plots constructed from serum sample measurements of the chicken serum samples, from which potential biomarkers were chosen. The ions furthest from the origin were discovered to most significantly distinguish both models and are the key metabolites that could completely separate the serum samples of the susceptible and resistant groups in Fig. 7. Moreover, these metabolites could be considered the key compounds involved in the pathogenesis of AS.

The loading plot displayed some of the potential resistant-AS-related metabolites on day 21 and 35, based on the variable importance in the projection (VIP) values. To identify these metabolites, we first searched the Human Metabolome Database (HMDB, http://www.hmdb.ca/), ChemSpider (http://www.chemspider.com/), and Kyoto Encyclopedia of Genes and Genomes (KEGG, http://www.kegg.com/) databases for candidates using mass and MS^E^ data. Because of the different possible mechanisms of fragmentation, items without mass fragment information were removed from the candidate list, and only the most probable were retained. A m/z value of 188.07 is shown as an example to illustrate the identification process. In addition to the base peak ion at m/z 188.0715, the ions at m/z 205.09 and m/z 227.07 were found in the positive ion spectrum (Fig. 7A). Thus, we inferred that the quasi-molecular ion was m/z 205.09 ([M + H]^+^) and the ions at m/z 227.07 and m/z 188.0715 were the fragment ions of [M + Na]^+^ and ([M + H − NH3]^+^). With different collision energy, the corresponding MS^E^ can be obtained (Fig. 7B). The ions at m/z 146.0607, m/z 118.0659 and m/z 91.0548 were found in the positive ion spectrum (Fig. 7B). To further define these structures, the molecular weight of 205.0964 Da was searched in the HMDB, Chemspider and KEGG database; then, the compounds without a phenyl group were removed from the candidates. In addition, samples were injected into the Q-TOF/MS under the same liquid chromatographic conditions and the accurate molecular weight of m/z 205.09 was found, which was the only peak in the extraction ion mass spectrum. This result was consistent with that of previous literature reports^14^. Finally, the substance was identified as tryptophan. Using the same method, other biomarkers on day 21 and 35 were identified. The identified biomarkers are summarized in Tables 1 and 2. Following the KEGG database search, 15 biomarkers were identified, which are primarily expressed among the pathways associated with phospholipid metabolism [lysoPC(18:1), PE(18:3/16:0), PC(20:1/18:3), DG(24:1/22:6/0:0), PS(18:2/18:0), PI(16:0/16:0), PS(18:0/18:1), PS(14:1/14:0)], inositol phosphate metabolism (dihydroxyacetone), bile acid metabolism (ursodeoxycholic acid), tyrosine tryptophan biosynthesis (tryptophan), pantothenate and CoA biosynthesis (L-valine), cycloserine, hypoxanthine, and 4-O-Methylmelleolide on day 21 (Table 1). We found upregulation of eight products of phospholipid metabolism, while other metabolites were found to be downregulated. Furthermore, the following 9 biomarkers (Table 2) were identified on day 35, LysoPC(18:0), LysoPE(20:1/0:0), LysoPC(16:0), LysoPE(16:0/0:0), hypoxanthine, dihydroxyacetone, 4-O-Methylmelleolide, LysoPC(18:2), and PC(14:1/22:1). Five of these metabolites were upregulated, whereas the others were suppressed. Taken together, these observations suggest that these metabolites likely have a critical regulatory function on metabolic alterations in low-temperature-induced AS chickens or potential biomarkers for resistant-AS.

**Table 1:** 15 Biomarkers of resistant-ascites syndrome detected using ultra-performance liquid chromatography-quadruple time-of-flight high-sensitivity mass spectrometry (UPLC Q-TOF/MS) in positive ion mode on day 21.

No	*t*_R__	Metabolite	m/z	Quasi-molecular ion	Formula	Molecular Weight	Trend^a^	Related Pathway
1	0.52	Dihydroxyacetone	203.0531	[M + Na] +	C_6_H_12_O_6_	180.0633	↓^***^	Pyruvic acid metabolism
2	9.16	Ursodeoxycholic acid	393.2978	[M + H]^+^	C_24_H_40_O_4_	392.572	↓^***^	
3	7.63	LysoPC(18:0)	524.3717	[M + H]^+^	C_26_H_54_NO_7_P	523.3637	↑	Lipid metabolism
4	0.59	Cycloserine	120.0813	[M + NH_4_]^+^	C_3_H_6_N_2_O_2_	102.0919	↓^***^	
5	0.59	Hypoxanthine	137.0461	[M + H]^+^	C_5_H_4_N_4_O	136.0385	↓^**^	
6	8.52	PE(18:3/16:0)	736.4517	[M + K]^+^	C_39_H_72_NO_7_P	697.9652	↑^***^	Lipid metabolism
7	8.81	PC(20:1/18:3)	810.5251	[M + H]^+^	C_46_H_84_NO_8_P	809.5934	↑^***^	Lipid metabolism
8	8.81	DG(24:1/22:6/0:0)	789.5466	[M + K]^+^	C_49_H_82_O_5_	751.1724	↑^***^	Lipid metabolism
9	8.81	PS(18:2/18:0)	788.5429	[M+H]^+^	C_42_H_78_NO_10_P	788.0432	↑^***^	Lipid metabolism
10	8.81	PI(16:0/16:0)	811.5286	[M+H]^+^	C_41_H_79_O_13_P	810.5258	↑^***^	Lipid metabolism
11	8.58	PS(18:0/18:1)	812.5402	[M+Na]^+^	C_42_H_80_NO_10_P	790.0591	↑^***^	Lipid metabolism
12	2.34	Tryptophan	188.0709	[M-NH_3_+H]^+^	C_11_H_12_N_2_O_2_	204.2252	↓^**^	
13	0.53	L-Valine	118.0865	[M+H]^+^	C_5_H_11_NO_2_	117.1463	↓^**^	
14	6.16	4-O-Methylmelleolide	437.1937	[M+Na]^+^	C_24_H_30_O_6_	414.4914	↓^*^	
15	7.75	PS(14:1/14:0)	678.4341	[M+H]^+^	C_34_H_64_NO_10_P	677.8464	↑	Lipid metabolism

^a^Change trend of resistant vs susceptible.

Levels of potential biomarkers were labelled with (↓) downregulated and (↑) upregulated. ^*^P < 0.05, ^**^P < 0.01, and ^***^P < 0.00.

**Table 2:** 9 Biomarkers of resistant-ascites syndrome detected by ultra-performance liquid chromatography-quadruple time-of-flight high-sensitivity mass spectrometry (UPLC Q-TOF/MS) in positive ion mode on day 35.

No	*t*_R__	Metabolite	m/z	Quasi-molecular ion	Formula	Molecular Weight	Trend^a^	Related Pathway
1	7.63	LysoPC(18:0)	524.3717	[M + H]^+^	C_26_H_54_NO_7_P	523.3637	↑^**^	Lipid metabolism
2	7.64	LysoPE(20:1/0:0)	525.3751	[M + NH_4_]^+^	C_25_H_50_NO_7_P	507.6408	↑^**^	Lipid metabolism
3	6.89	LysoPC(16:0)	496.3404	[M + H]^+^	C_24_H_50_NO_7_P	495.6301	↑^*^	Lipid metabolism
4	0.52	Dihydroxyacetone	203.0531	[M + Na]^+^	C_6_H_12_O_6_	180.0633	↓	Pyruvic acid metabolism
5	6.16	4-O-Methylmelleolide	437.1937	[M + Na]^+^	C_24_H_30_O_6_	414.4914	↓	
6	0.59	Hypoxanthine	137.0461	[M+H]^+^	C_5_H_4_N_4_O	136.0385	↑	
7	6.76	LysoPC(18:2)	520.3402	[M+H]^+^	C_26_H_50_NO_7_P	519.6515	↓^*^	Lipid metabolism
8	7.00	LysoPE(16:0/0:0)	454.2929	[M+H]^+^	C_21_H_44_NO_7_P	453.5503	↑^**^	Lipid metabolism
9	8.59	PC(14:1/22:1)	808.5821	[M+Na]^+^	C_44_H_84_NO_8_P	785.5934	↓	Lipid metabolism

^a^Change trend of resistant vs susceptible.

Levels of potential biomarkers were labelled with (↓) downregulated and (↑) upregulated. ^*^P < 0.05, ^**^P < 0.01, and ^***^P < 0.001.

**Figure 8:**
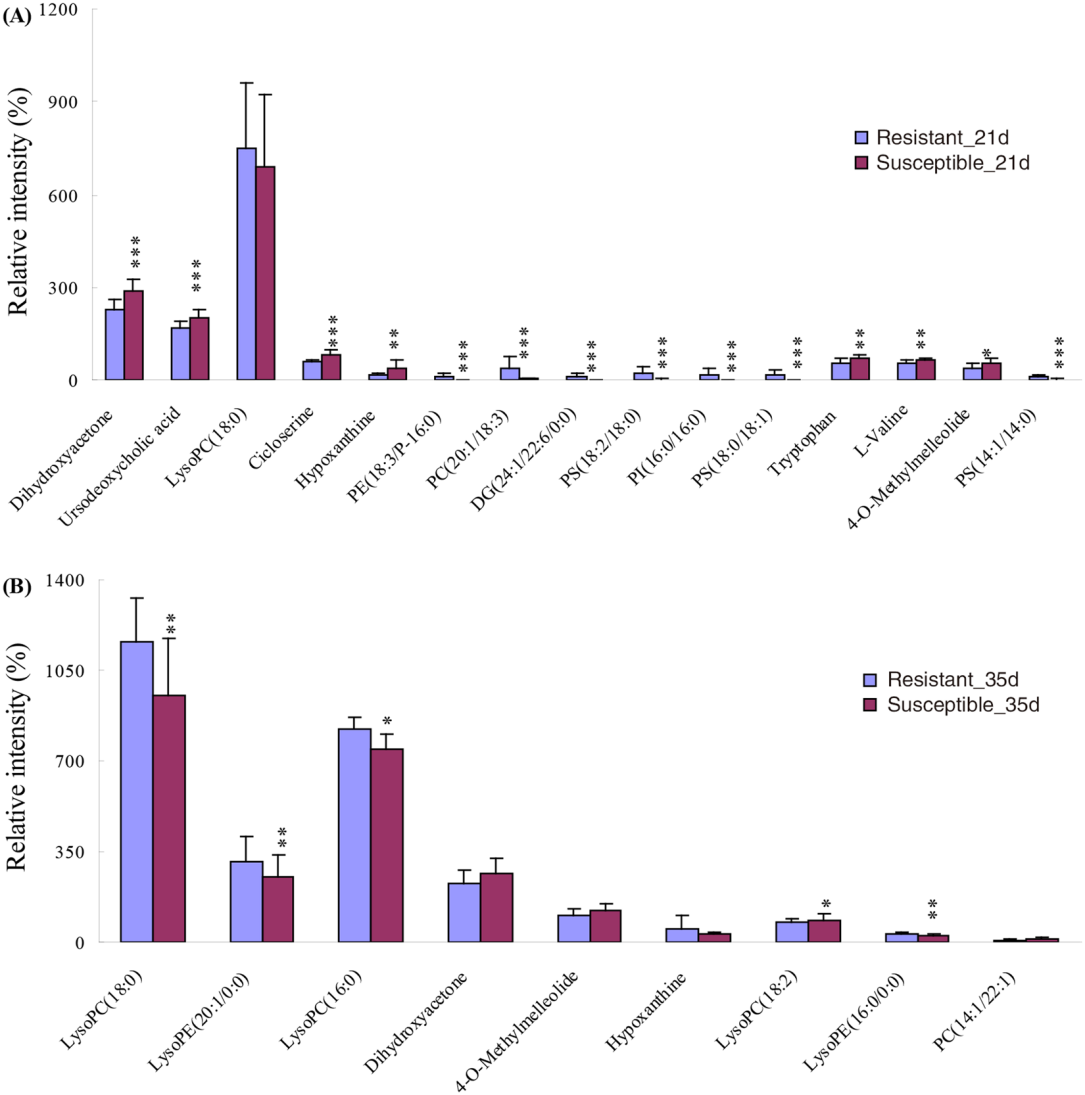
Comparison of the relative intensity of putative potential biomarkers in the resistant group and susceptible group on day (**A**) 21 and (**B**) 35.

**Figure 9:**
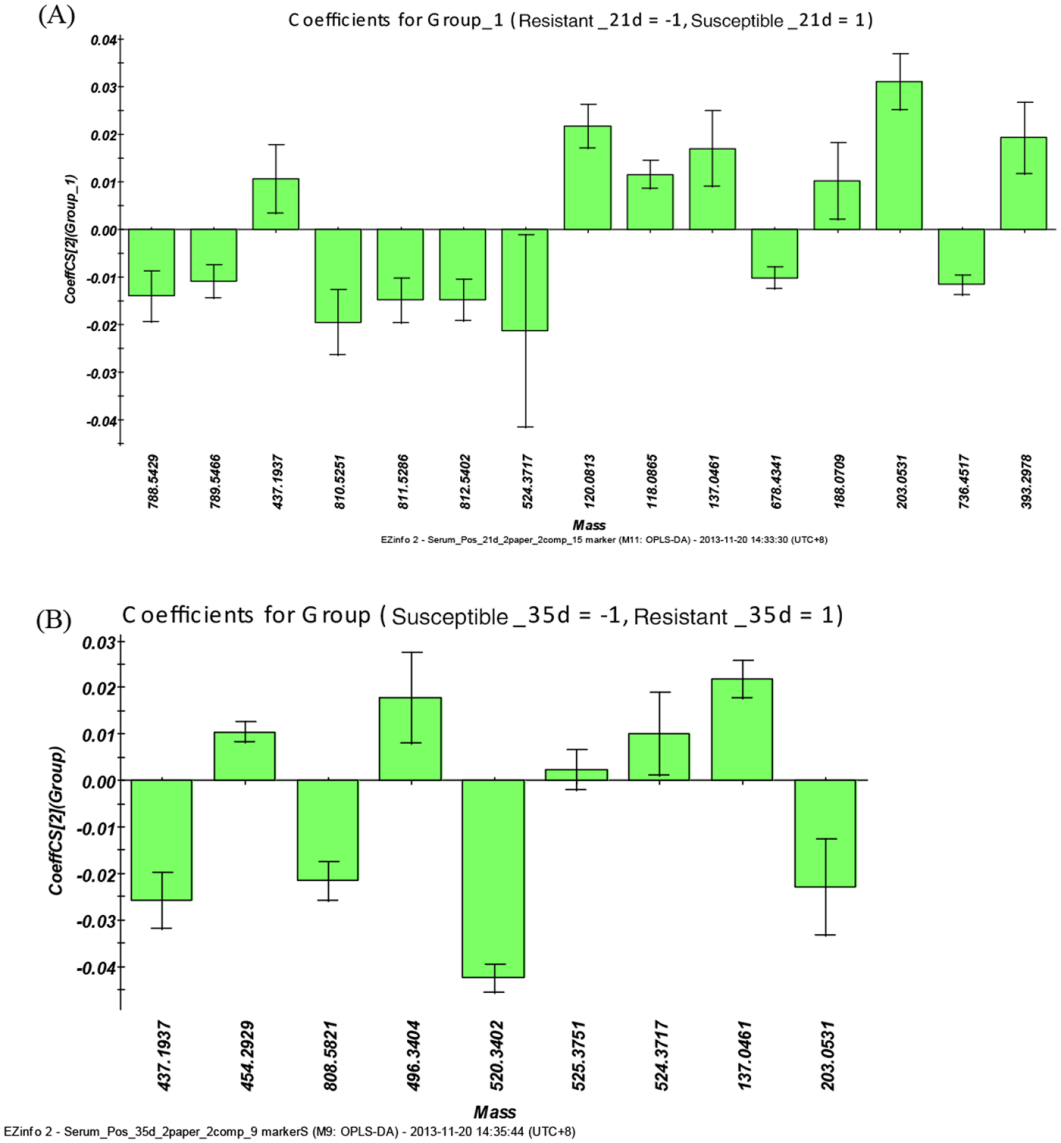
OPLS-DA loading plot in positive ion mode from the resistant group and susceptible group on day (**A**) 21 and (**B**) 35. The loading plots represent which metabolites are quantitatively higher or lower in suspectible group compared with resistant group.

Figure 8 shows the relative concentrations of the biomarkers identified in the resistant and susceptible groups on day 21 and 35, and the specific changes in identified biomarkers between the groups were reflected. Figure 9 shows the loading diagram correlation coefficients for the identified potential biomarkers. The absolute value of the coefficient represents the importance of the corresponding metabolite; in other words, the importance of the PLS-DA analysis results could indirectly influence the differences in the corresponding metabolites of the two groups. As shown in Fig. 9, the first four substances sorted by absolute values of correlation coefficients were LysoPC(18:0), dihydroxyacetone, cycloserine and PC(20:1/18:3) on day 21, and the first three were LysoPC(18:2), 4-O-Methylmelleolide and dihydroxyacetone on day 35; among these substances, dihydroxyacetone were equally important at both time point.

**Figure 10:**
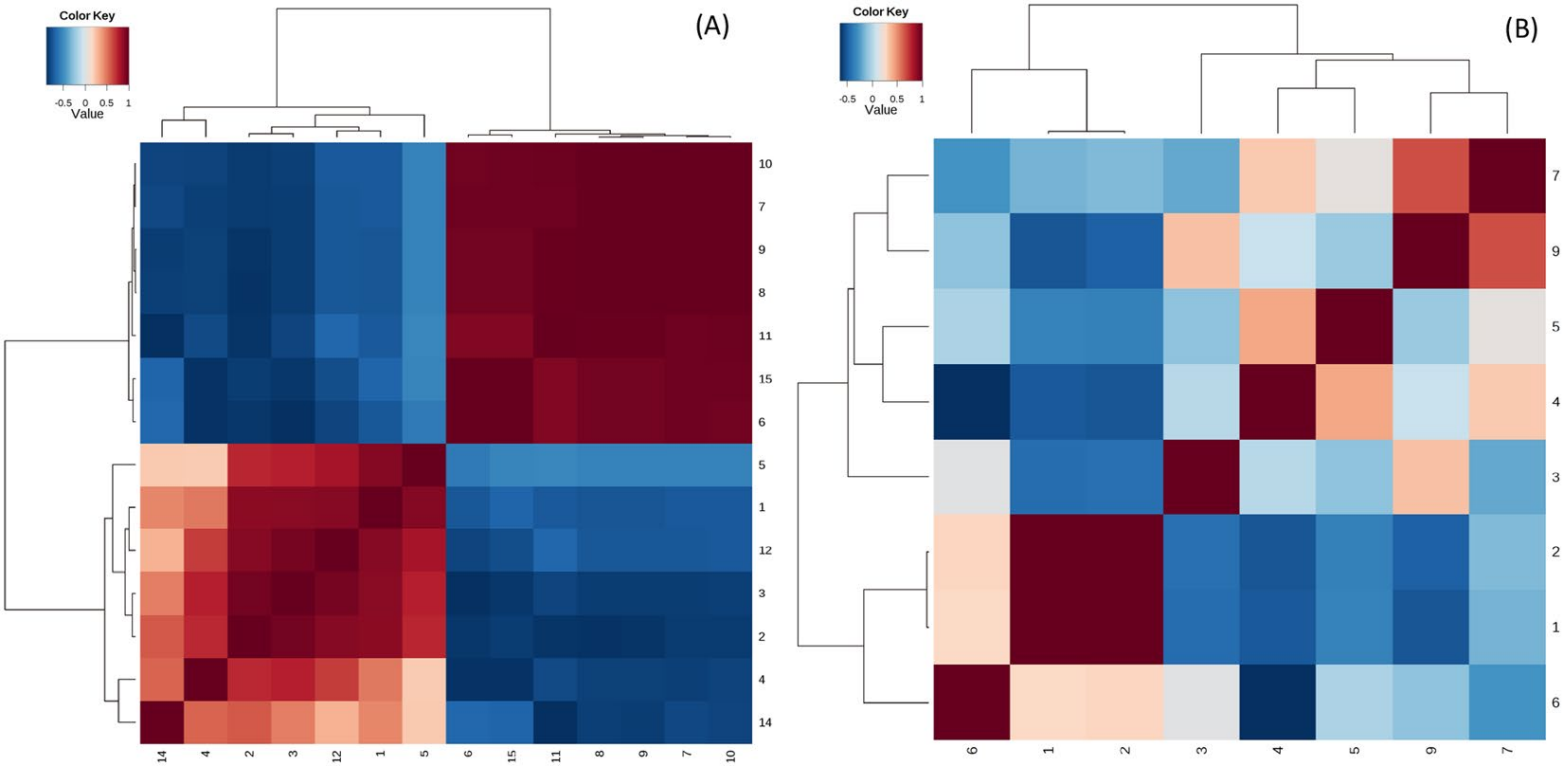
Correlation analysis of the differential metabolites on day (**A**) 21 and (**B**) 35 are marked on the S-plots. Numbers consist with Tables 1 and 2.

**Figure 11:**
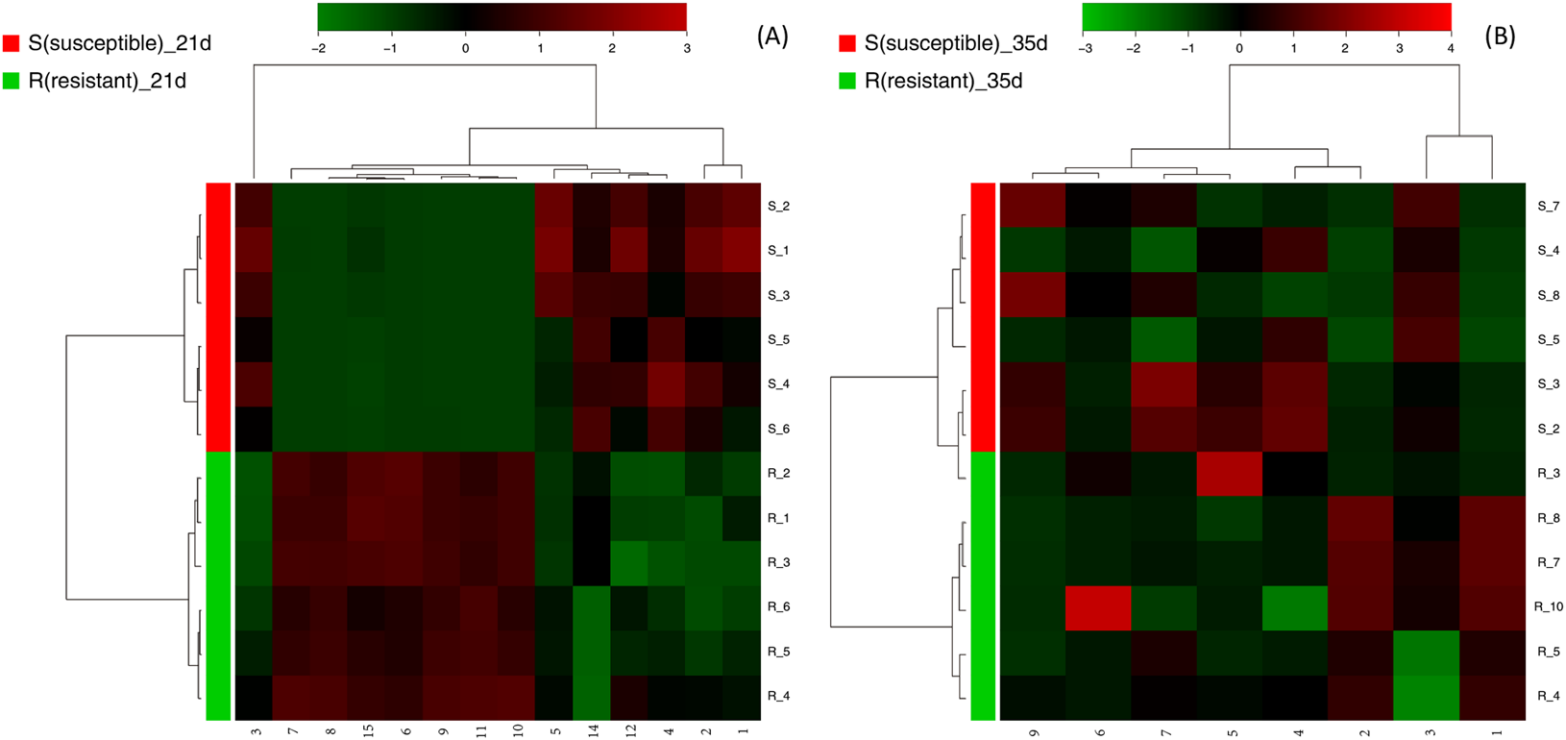
Heatmaps for serum metabolites on day (**A**) 21 and (**B**) 35. Colour of each section is proportional to significance level of change in metabolites (red, upregulated and green, downregulated). Rows: samples; Columns: metabolites; Numbers consistent with Tables 1 and 2.

The correlation plot analysis of the differentially accumulated metabolites (Fig. 10) and the heatmap visualization (Fig. 11) show distinct segregation between the resistant and susceptible groups. These analyses differentiate the resistant-AS chickens by adjusting multiple metabolic pathways away from the AS birds. The heatmap was constructed based on the importance of the potential candidates. Based on the plots, the metabolites that distinguished the resistant group from the susceptible group were considered potential biomarkers.

### Transcriptomic Alterations Induced by AS and resistant-AS

**Figure 12:**
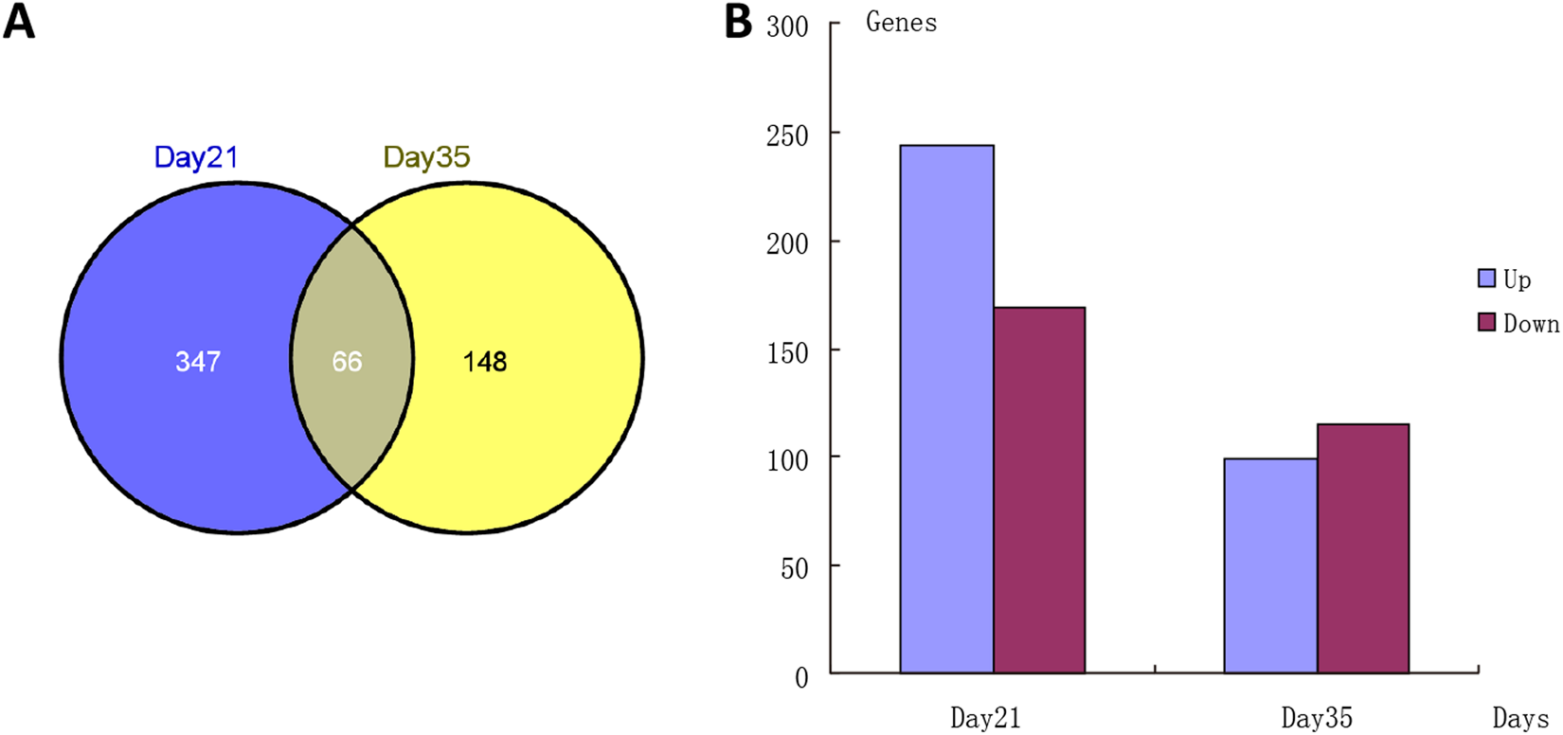
Comparison of differentially expressed genes (DEGs) in resistant and susceptible groups. Number of (**A**) DEGs and (**B**) upregulated and downregulated DEGs.

As shown in Fig. 12, compared to the susceptible group, 413 genes were discovered to be DEGs, with 244 and 169 showing enhanced and suppressed expression in the resistant group on day 21. On day 35, 214 genes were identified as DEGs, with 99 and 115 showing enhanced and suppressed expression in the resistant group.

**Figure 13:**
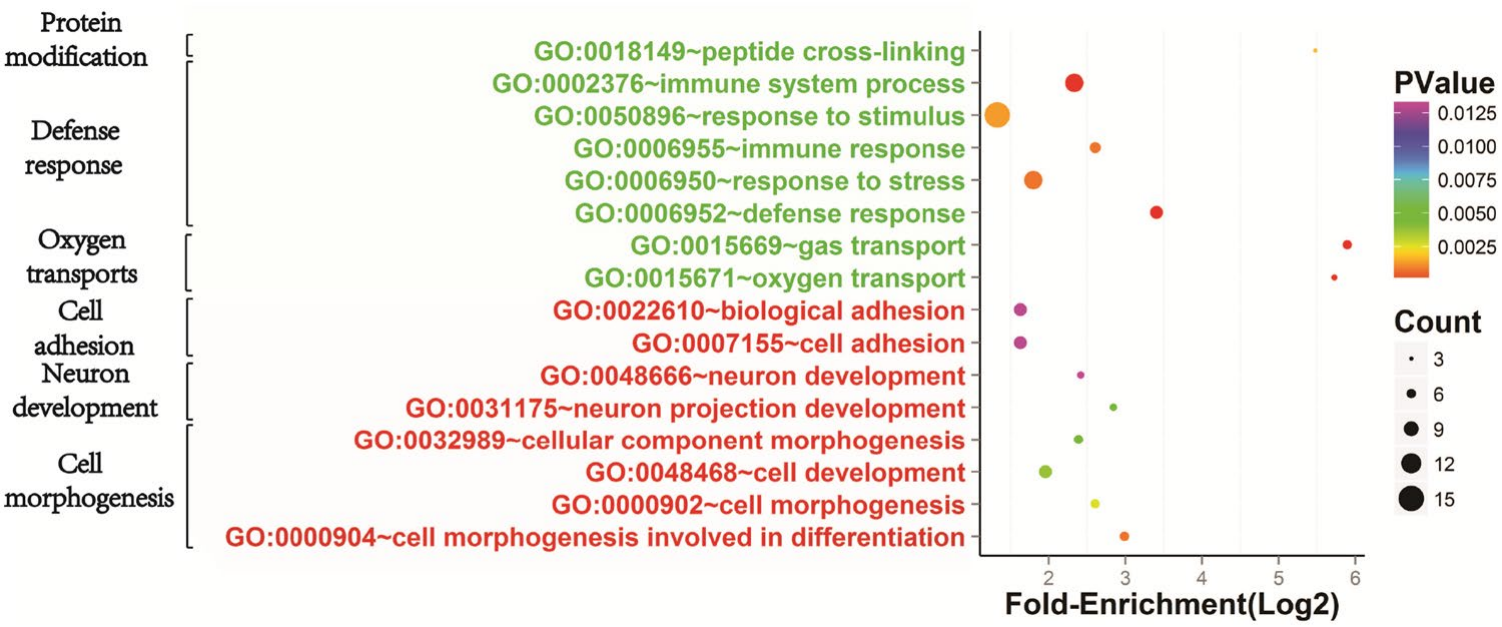
Gene ontology (GO) analysis of differentially expressed genes (DEGs) in resistant group compared to susceptible group on day 35. Circle represents enrichment significance of DEGs. Circle area represents number of DEGs followed in each category. Green and red colour of GO name indicates downregulation and upregulation, respectively.

As shown in Fig. 13, GO analysis filtered by fold enrichment >2, P < 0.05, and a false discovery rate (FDR) <20% showed that these DEGs primarily belonged to the suppressed oxygen transportation (GO:0015671~oxygen transport and GO:0015669~gas transport), defensive reactions (GO:0006952~defense response, GO:0006950~response to stress, GO:0006955~immune response, GO:0050896~response to stimulus, and GO:0002376~immune system process), and protein modifications (GO:0018149~peptide cross-linking). Furthermore, the DEGs were upregulated for cell morphological formation (GO:0000904~cell morphogenesis involved in differentiation, GO:0000902~cell morphogenesis, GO:0048468~cell development, and GO:0032989~cellular component morphogenesis), neural development (GO:0031175~neuron projection development and GO:0048666~neuron development), cell adhesion (GO:0007155~cell adhesion and GO:0022610~biological adhesion), and the TGF-beta signalling pathway (gga04350:TGF-beta signalling pathway). Among these, the GO:0050896~response to stimulus had the most enrichment genes (n = 15), while GO:0015669~gas transport had the highest fold-enrichment (59.64) and GO:0015669~gas transport had the lowest P value for fold-enrichment (*P* = 0.0000000236) on day 21. Figure 13 also shows that the DEGs mainly belonged to down-regulated oxygen transportation (GO:0015669~gas transport, GO:0015671~oxygen transport) on day 35. The DEGs that were enriched in oxygen transport function on days 21 and 35 were the down-regulated genes including Hemoglobin zeta (HBZ), Hemoglobin alpha 2 (HBAD), Hemoglobin alpha 1 (HBAA) and Rh-associated glycoprotein (RhAG) (Table 3).

**Table 3:** The DEGs related to oxygen transport in the resistant group compared to susceptible group on day 21 and 35.

Gene symbol	Gene title	Fold-change		Main biological functions
		21d	35d	
HBZ	haemoglobin, zeta	−5.78	−4.46	Red cell maturation and negative regulation of transcription from RNA polymerase II
HBAD	haemoglobin, alpha 2	−3.90	−2.73	Participates in the oxygen transfer from the lungs to the surrounding tissues
HBAA	haemoglobin, alpha 1	−2.69	−2.11	Participates in the oxygen transfer from the lungs to the surrounding tissues
RhAG	Rh-associated glycoprotein	−3.02	−2.05	Participates in the activity of ammonium transmembrane transport, as a CO_2_ gas channel

Fold-changes represent relative gene expression levels.

**Figure 14:**
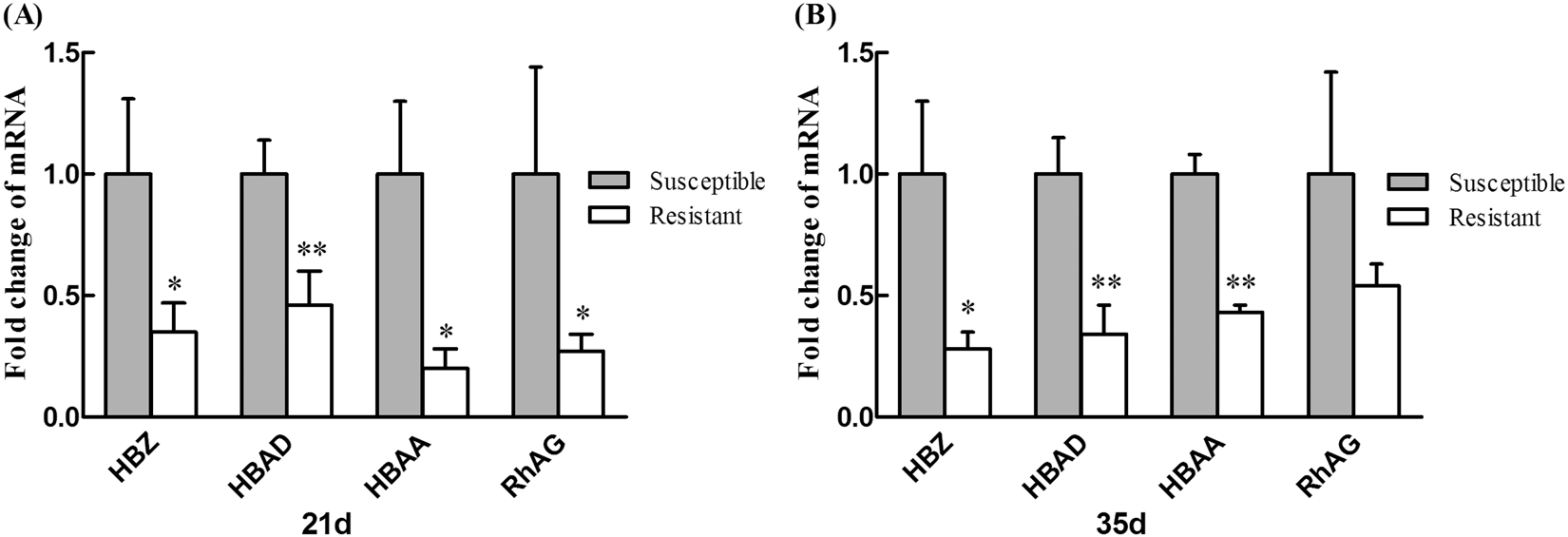
Validation of Hemoglobin zeta (HBZ), Hemoglobin alpha 2 (HBAD), Hemoglobin alpha 1 (HBAA) and Rh-associated glycoprotein (RhAG) mRNA expression in the resistant group and susceptible group on day (A) 21 and (B) 35. Data are mean ± standard deviation (SD). **P < 0.01 and *P < 0.05.

Figure 14 shows the hepatic expression of HBZ, HBAD, HBAA and RhAG mRNA on days 21 and 35. Compared to the susceptible group, the expression of HBZ, HBAA and RhAG mRNA was significantly decreased ((Fig. 14A, P < 0.05), and the expression of HBAD mRNA was extremely significantly decreased (Fig. 14A, P < 0.01) in the resistant group on day 21. On day 35, the expression of HBZ mRNA was significantly decreased ((Fig. 14B, P < 0.05), and the expression of HBAD and HBAA mRNA was extremely significantly decreased (Fig. 14B, P < 0.01) in the resistant group.

## Discussion

One of the mechanisms underlying the pathogenesis of AS is metabolic hypoxia or low oxygen utilization efficiency^15^. Under hypoxic conditions, the compensatory increase of the heart and liver, which are known to be the major users of oxygen in animals, was regarded as the judgment index in previous studies of AS, and AHI > 0.27 is a commonly-used indicator. The body weight of AS broilers was significantly lower than that of normal broilers, and the liver index, heart index and AHI were significantly increased, which reflected the enlargement of the important organs and the right heart of AS^16^. AS broilers are often in a subclinical state because of the presence of hypoxia in these organisms, which is accompanied by changes in routine blood indices, such as increased HGB and HCT^17, 18^. The changes in these indicators imply that the ability of blood to transport oxygen was increased in a compensatory manner in broilers. Meanwhile, the changes in the blood parameters could cause increased blood viscosity^19–21^, increased erythrocyte fragility, pulmonary hypertension and vascular remodelling^22^, eventually leading to AS.

Pulmonary vascular remodelling is an important pathological feature of pulmonary hypertension. In the study of broilers, it was observed that pulmonary vascular remodelling, which is mainly characterized by thickening of the pulmonary artery smooth muscle layer, occurs during the development of AS. With endothelial cell injury or hemodynamic changes induced by low temperature, the function of endothelial cells as a physiological barrier and the connection of endothelial cells with the musculocutaneous of smooth muscle cells led to uncontrolled proliferation and matrix synthesis^23, 24^. In this present study, the AHI and HCT values, as well as the thickness of the pulmonary artery in the susceptible group, were significantly higher than those in the resistant group were. These significant differences between the susceptible and resistant groups illustrated that the AS and resistant-AS models were successfully developed for use in this study.

In this present study, 15 biomarkers associated with AS were found on day 21, which were mainly distributed among the pathways related to lipid metabolism, protein metabolism and glucose metabolism. 9 biomarkers associated with AS were found on day 35 and were mainly distributed among the pathways related to lipid metabolism and glucose metabolism.

Eight (all up-regulated) of the 15 metabolic differences on day 21 and six (all up-regulated except LysoPC (18:2) and PC (14:1/22:1)) of the nine metabolic differences on day 35 were related to phospholipid metabolism, accounting for 53.33% and 66.67% respectively. From the data, it can be concluded that abnormalities in phospholipid metabolism play an important role in the occurrence of AS. Phospholipids are lipid compounds that contains phosphate in their molecular structures. They are widely distributed in animal brain, bone marrow, liver, heart, kidney, lung, blood, milk, and eggs, as well as in most seed plants and some microorganisms, so almost all biological cells contain phospholipids. Phospholipids can be divided into glycerol phospholipids and sphingolipids based on their molecule structures, which contain different alcohols. The glycerol phospholipids include phosphatidylcholine (PC, commonly known as lecithin), phospholipid acyl ethanol amine (PE, commonly known as brain phospholipid), phosphatidyl inositol (PI, commonly known as inositol phospholipid), phosphatidylserine (PS), phosphatidylglycerol (PG), phospholipid phosphatidylglycerol (DG, commonly known as cardiolipin), and acetal phospholipids. The main sphingolipid is sphingomyelin^25^. Phospholipids are not evenly distributed on cell membrane, with PC and SM usually located on the outer membrane and PE and PS located on the inner membrane^26, 27^. PS is the only phospholipid that can regulate the functional status of key proteins in the cell membrane^28^. The main role of PS in signal transduction is transferring the protein kinase C (PKC) to the cell membrane through the specific connection of PKC and PS, and the activities of Ca^2+^-independent PKCs are all strictly dependent on PS. Moreover, PS can control the activity of other enzymes involved in signal transduction processes, such as diacylglycerol kinase, C-Paf-1 proteinase and nitric oxide synthase^29^. In the early stage of apoptosis, PS and PE can move from the inner side of the cell membrane to the cell membrane surface, where they are exposed to the extracellular environment, leading to their identification and phagocytosis by macrophages. A loss of PS and PE and the asymmetry of the cell membrane are all early signs of apoptosis^30–33^. Ackerstaff *et al*.^34^ and Glunde *et al*.^35^ believed that an increased level of cholinergic metabolites, such as PC, LysoPC, and PE, in tumour tissues was closely related to the proliferation of cancer cells. Due to the rapid proliferation of tumour cells, they consume a large amount of phosphate metabolites, which can lead to a significant reduction of these metabolites in the blood. In the present study, it was found that in broilers in the induced early resistance group, the levels of phospholipid compounds, such as PS, PE, PI, DG, and LysoPE, in the serum were significantly higher than those in broilers in the susceptible group, indicating that broilers in the resistance group had better resistance to apoptosis and a better antioxidant function than those in the susceptible group.

Tryptophan is one of the essential amino acids in animals and cannot be synthesized, so it must be taken in from the diet. There are two main sources of tryptophan for animals. Approximately two-thirds of the tryptophan in an animal’s body is endogenous tryptophan derived from the decomposition of tissue proteins; the other one-third of the tryptophan in an animal’s body is exogenous tryptophan that is digested and absorbed from the diet. There are also two pathways of tryptophan metabolism: one is used to synthesize protein and the other metabolizes proteins through decomposition. The tryptophan that an animal ingests is mainly used for *in vivo* protein synthesis, but is also involved in many other internal physiological processes^36^. Tryptophan is involved in animal protein synthesis, as follows: First, tryptophan is activated by tryptophan tRNA synthetase and is then combined with the corresponding tRNAs to form a Trp-tRNA mixture, which can be used to synthesize tissue proteins and avoid amino acid degradation. The decomposition of tryptophan occurs as follows: 1) The main degradation pathway involves turning tryptophan into canine urinary amino acid via Tryptophan-2,3-dioxygenase, followed by a series of pathways to generate nicotinic acid, acetyl CoA, alanine and other products and is the main tryptophan degradation pathway; 2) the second pathway involves producing serotonin through tryptophan hydroxylase and decarboxylase, although the proportion of this pathway in the whole tryptophan degradation process is relatively low. 3) Tryptophan can also be turned into pyruvate by the action of ammonia removal, and the latter can be turned into heteroauxin by removing the carboxyl, which is then excreted in urine. Acetyl CoA can enter the tricarboxylic acid cycle to participate in the energy supply and can form body fat or participate in the metabolism of other substances, while alanine can produce both acetyl CoA and sugar. Nicotinic acid is a precursor for the synthesis of NAD and NADP, both of which participate in redox reactions. Figure 14 lists the pathways of tryptophan metabolism in animals. This study found that the blood tryptophan level was decreased in 21-day-old broilers in the resistant group, probably because the broilers in the susceptible group needed a large amount of tryptophan to decompose it to CoA and thus to supply energy. Insufficient substrates of the tricarboxylic acid cycle results in reduced ATP synthesis, leading to the degradation of amino acids and inhibition of protein synthesis^37^.

Dihydroxyacetone, which is an intermediate product of glucose metabolism in animals, has an important role in the metabolism of the organism. The metabolism of dihydroxyacetone in organisms is complex. Dihydroxyacetone is an important intermediate that links glucose metabolism to lipid metabolism, allowing it to effectively regulate the relationship between glucose metabolism and lipid metabolism. In the present study, the serum dihydroxyacetone level in the resistant group was decreased on days 21 and 35, likely because the phosphorylation of dihydroxyacetone generated dihydroxyacetone phosphate for oxidation energy in susceptible broilers, resulting in a high blood level.

Haemoglobin (Hb) is a red oxygen-carrying protein containing ferrous ions that is widely present in leguminous plants, in the blood of vertebrates, and in a small number of invertebrates. Hb is composed of four chains, two α chains and two β chains, each of which has a cyclic heme that can contain a bivalent iron ion. The binding of heme and oxygen generates oxyhemoglobin, which is bright red. After dissociation, a pale blue colour appears. Hb can bind and transport oxygen and can also bind carbon dioxide, carbon monoxide, and cyanide and shows a certain biological characteristic^38^. There are seven types of Hb in chickens: HbA, HbD, HbP, HbM, HbE, HbL and HbH. HbA and HbD are the primary and secondary haemoglobins of adult individuals, respectively. HbP, HbM, and HbE are primary and secondary haemoglobins, while HbL and HbH are secondary haemoglobins in the early stage of fetal^39^. Red blood cells are the carriers of haemoglobin, and there is a direct proportional relationship between them^40^. In this present study, the HCT values in the susceptible group were significantly higher than those in the resistant group on days 21 and 35, which was consistent with the results that showed that the expression levels of HBZ, HBAD, and HBAA in the susceptible group were higher compared to those in the resistant group.

RhAG (Rh-associated glycoprotein, also known as Rh50) is a glycosylated protein composed of 409 amino acids and crosses the red cell membrane 12 times. Studies have shown that the Rh complex may be involved in the connection of the membrane phospholipid bilayer to the cytoskeleton, helping to maintain the normal structure of the cell; it may also be used as a CO_2_ gas channel because it functions as an ammonium transport channel^41, 42^.

In 2002, Soupene and other scholars researched Rhl (Rh-like glycoprotein) of green algae and found that it was regulated by CO_2_. Rh1 is up-regulated when the green algae are cultured with 3% CO_2_ or air with a high CO_2_ concentration; while it is down-regulated when algae are cultured in normal air or when the high CO_2_ concentration is changed to normal air. These findings indicate that RhAG has an important function in CO_2_ transport^43^. In 2006, Endeward and other scientists determined the CO_2_ permeability of different groups in normal and deficient erythrocyte membranes and found that when the oxygen permeability rate of normal red blood cells is close to 0.15 cm/s, the permeability of red blood cells that lack the Rh protein complex is reduced, while groups that have defects of other membrane proteins showed a normal CO_2_ permeability. This suggests that the Rh protein has an important role in the CO_2_ permeability of erythrocyte membranes. Therefore, Endeward *et al*. suggested that the RhAG protein functions as a CO_2_ gas channel^44^. In the present study, the expression of RhAG in the susceptible group was significantly higher than that in the resistant group on days 21 and 35, which may be because RhAG serves as a CO_2_ gas channel, and a higher concentration of CO_2_ would cause a higher expression of RhAG, so the oxygen concentration was relatively lower than that of the resistant group. These observations also further illustrated that metabolic hypoxia or a low oxygen utilization efficiency were important factors associated with the induction of AS in broilers^15^.

## Conclusions

We observed significant differences in the metabolomes in the serum and transcriptomes in the liver of broiler chickens with low-temperature-induced AS and resistant-AS, confirming that these models are useful for investigating the molecular mechanisms underlying the development of resistant-AS in chickens. Furthermore, the integrative analysis revealed that glycerophospholipid metabolism plays an important role in the development of resistant-AS broilers. This study has provided integrative and systematic insights into the molecular basis of the development of resistant-AS.

## Methods

### Ethics Statement

This study was carried out in strict compliance with the Guidelines for Experimental Animals established by the Ministry of Science and Technology (Beijing, China). All the experimental protocols were approved by the Science Research Department (in charge of animal welfare) of the Poultry Institute, Chinese Academy of Agricultural Sciences (CAAS) (Yangzhou, China).

### Experimental Design and Animal Management

Male 108-day-old meat-type chickens (Ross-308) were purchased from a local broiler breeder and hatchery farm (Zhengda Broiler Development Center of China Agriculture University, Hebei, China) and were divided into six replicates of 18 broilers each. The average initial body weight was 47.74 ± 0.55 g. The chickens were bred in cages (2.4 × 0.6 × 0.6 m) with screen-wired floors. The temperature in the chicken room was maintained at 32–35 °C during the first week and was then decreased by 1 °C every other day until a final temperature of 27 °C was reached on day 13. To increase their susceptibility to AS, the birds were exposed to AS induction conditions, consisting of 17 °C during the day and 14 °C at night, commencing on day 14^22, 45^. The broilers were provided water freely, fed a commercial broiler diet, and maintained on a daily 23-h fluorescent lighting cycle during the entire study. The diets were prepared to fulfill or exceed all nutritional requirements^46^ and were administered as pellets.

### Sample Collection

On days 21 and 35, before the syndrome peak on day 39^4, 47^, three birds were selected from each replicate (18 birds per group) and weighed after an 8-h fast. During necropsy, samples of the venous blood were collected from the wings for haematological and metabolomic analyses. To measure the HCT level, 1.5-mL blood samples were withdrawn from each bird and placed into an ethylenediaminetetraacetic acid (EDTA)-K3 anticoagulation tube (evaluated using a Sysmex KX-21N Automatic Blood Analyzer, Kobe, Japan). Additional 1.5-mL blood samples were separated by centrifugation at 3,500 × *g* for 10 min, the serum was subsequently collected, placed in a freezing tube, snap-frozen in liquid nitrogen, and then stored at −80 °C for the metabolomic analyses.

Following the collection of blood samples, the birds were euthanized by jugular bleeding, the hearts were dissected out, and the right and total ventricular weight of each heart were measured to estimate the AHI^48^. Based on these results, we chose six birds each that were AS-positive and AS-insensitive and determined to be susceptible (susceptible group) and resistant (the resistant group). The determination criteria were based on the following three factors: (1) a bird was considered resistant if it had HCT < 0.36 and AS-positive if it showed HCT ≥ 0.36; (2) an AHI < 0.28 indicated a bird was resistant while values ≥ 0.28 were considered to confirm AS-positive status; and (3) the absence of yellow liquid in the abdominal cavity and pericardium indicated a bird was resistant while it was considered to be AS-positive if there was >10 mL of yellow liquid in the abdominal cavity and pericardium. The serum of susceptible and resistant chickens (six each) were selected for the metabolomic profile analysis while the liver tissues of susceptible and resistant chickens (three each) were selected for the transcriptomic analysis and gene expression analysis.

### Histopathological Examination

To evaluate the histopathological changes in the pulmonary arteries of broilers with AS, bronchi-adjacent tissue segments (0.5-cm thick) were removed from the lungs, fixed with 10% buffered formalin for >24 h, and then dehydrated using an increasing ethanol gradient. The lung tissue sections were treated with dimethylbenzene until they turned transparent, paraffin-embedded, cut into 5-μm thick sections, and then Weigert-Van Gieson stained for elastin^49^. Small pulmonary arterioles with external diameters of 20–50, 50–100, and 100–200 μm were examined under a BH2 Olympus microscope (DP71, Olympus, Tokyo, Japan) using the advanced software (Motic Images Advanced 3.0). Then, 12 cross-sectional areas were selected. The adventitia and lumen diameters were measured to analyse the RMT (%). The RMT of the pulmonary arterioles with different cutting angles under conditions of either contraction or relaxation were calculated from the above measurements based on previously reported methods^50, 51^.

### Metabolomic Sample Preparation

The 12 serum samples (100 μL each) were vortexed for 2 min in 500 μL of a methanol/acetonitrile mixture (1:9, v/v, refrigerated at 4 °C for 30 min beforehand) in an ice bath. After centrifugation at 18,500 × *g* at 4 °C for 15 min, 300 μL of the supernatant was collected and analysed using UPLC. The extracted livers were immediately washed with physiological saline, and then 100 mg of each liver tissue sample was shredded, homogenized in physiological saline (2-fold volume), and stored at −80 °C. Before the subsequent analysis, the liver samples (100 μL) were thawed at 4 °C and vortexed for 5 min in 250 μL of acetonitrile (refrigerated at 4 °C for 30 min beforehand) in an ice bath. After centrifugation at 18,500 × *g* at 4 °C for 15 min, 150 μL of the supernatant was collected and analysed using UPLC.

### Chromatographic Separation

The UPLC analysis was performed using a Waters Acquity Ultra Performance LC system (Waters, Milford, MA) equipped with a Waters SYNAPT G2 Q-TOF HDMS (Waters MS Technologies, Manchester, UK). The chromatographic separation was carried out at 45 °C using an ACQUITY UPLC BEH C_18_ column (2.1 mm × 100 mm, 1.7-μm, Waters MS Technologies, Manchester, UK). We used a mobile phase consisting of water (A) and acetonitrile (B), each with 0.1% formic acid. The UPLC elution of the serum samples was performed using the following optimized conditions: 0–1.0 min, 1–5% B; 1.0–3.0 min, 5–20% B; 3.0–8.0 min, 20–85% B; 8.0–10.0 min, 85–99% B; 10.0–11.0 min, 99% B; and 11.0–12.0 min, 99–1% B. In addition, the liver samples were eluted using the following optimized UPLC conditions: 0–0.5 min, 1% B; 0.5–2.0 min, 1–50% B; 2.0–9.0 min, 50–99% B; 9.0–10.0 min, 99% B; and 10.0–12.0 min, 99–1% B. We used a 0.45 mL/min flow rate, the autosampler was maintained at 4 °C, and the sample injection volume was 2 µL for each run.

### MS

The MS analysis was conducted using an SYNAPT G2 Q-TOF HDMS (Waters MS Technologies, Manchester, UK), consisting of a quadrupole and orthogonal acceleration time-of-flight tandem mass spectrometer. The scan range of the molecular weight was 50–1200 m/z. The capillary and cone voltages for the positive electrospray mode were set at 3.2 kV and 45 V, respectively while the corresponding settings for the negative electrospray mode were 3.0 kV and 40 V, respectively. The other conditions were as follows: desolvation gas, 800 L/h at 450 °C; cone gas, 50 L/h; and source temperature, 120 °C. The MS was performed at a resolution of 25,000 using dynamic range extension. The data was acquired at a rate of 0.2 s. All the analyses were carried out using the lockspray to guarantee the precision and reproducibility. Leucine-enkephalin ([M + H]^+^ = 556.2771, [M − H]^+^ = 554.2615) was used as the lockmass at a concentration of 200 ng/mL and a flow rate of 5 μL/min. The data were acquired in the continuum mode using a lockspray frequency of 10 s, and the mean of 10 scans was obtained. All the data collection and analyses were monitored using the Waters MassLynx v4.1 software.

### RNA Preparation

The total RNA was extracted from liver samples of three chickens from each of the two groups (total of six samples) using Trizol (Invitrogen, Carlsbad, CA. USA), strictly following the instructions of the manufacturer. Following the RNA extraction, DNase I (Ambion, Austin, TX) digestion was conducted, and the RNA concentration and purity were analysed by measuring the absorbance at 260 nm and subsequently calculating the A260/A280 ratio using a NanoDrop ND-2000 spectrophotometer (Nano-drop Technologies, Wilmington, DE, USA). The total RNA was stored at −80 °C until further use.

### cDNA Library Preparation and Sequencing using RNA-Seq

To enrich the mRNA, two rounds of hybridization to oligo (dT) beads (Invitrogen, Carlsbad, CA, USA) were performed on the total RNA (7 µg) of each sample. Ribosomal RNA (rRNA) contamination was analysed using an RNA picochip with a BioAnalyzer (Agilent, Santa Clara, CA). The mRNA generated was used to establish cDNA libraries using an RNA-seq sample preparation kit (Illumina, San Diego, CA, USA). Six samples were sequenced separately using an Illumina HiSeq2000 with a 100-bp pair-end read length.

### Quantitative Real-time PCR of Gene Expression

Quantitative real-time PCR (RT-PCR) was performed to validate the 4 DEGs mRNA using StepOnePlusTM (Applied Biosystems by Life Technologies, USA). The design and synthesis of the primers by Invitrogen Biotechnology Co., Ltd. (Shanghai, China) were described in Table 4.

**Table 4:** Primer sequences for the target genes.

Gene	GenBank number	Primer position	Primer sequence (5′ → 3′)	Product size (bp)	Reference
GAPDH	NM_204305	Forward	GGTGAAAGTCGGAGTCAACGG	108	Druyan *et al*.^56^
		Reverse	CGATGAAGGGATCATTGATGGC		
HBZ	NM_001004374.2	Forward	AAGGTGGCTACCCAGATTGA	121	this study
		Reverse	GAAGCTGAACTGAGCCTTGG		
HBAD	NM_001004375.2	Forward	CCCGTCAATTTCAAGCTGTT	117	this study
		Reverse	AGACACGGCAGACAGGAACT		
HBAA	NM_001004376.3	Forward	GACCCTGGAAAGGATGTTCA	116	this study
		Reverse	AGGCAGCCACTACCTTCTTG		
RhAG	NM_204464	Forward	GAGCCTCAATGACCATCCAT	129	this study
		Reverse	TGGCAAATATGTCCGAGTGA		

Standard curves and melting curves of each primer pair were implemented to insure amplification specificity for each gene. Glyceraldehyde-3-phosphate dehydrogenase (GAPDH) was used as a reference gene. The 20 μL PCR reaction system contained 10 μL SYBR^®^ Premix Ex Taq (Tli RNaseH Plus) (2×) (TaKaRa Biotechnology Co., Ltd., Dalian, China), 0.4 μL of the forward and reverse primer for each gene, 0.4 μL ROX Reference Dye (50×) (TaKaRa), 2 μL of RT product and 6.8 μL dH_2_O. The PCR conditions consisted of 30 s initial denaturation at 95 °C, followed by a two-step amplification program (5 s denaturation at 95 °C, 30 s annealing/extension at 60 °C) repeated 40 cycles. The 2^−ΔΔCt^ method was used to analyse mRNA abundance. All of the samples were analysed in triplicate and the average values of these measurements were used to calculate the expression of mRNA.

### Data Analysis

After importation of the original MS data into the Markerlynx XS (Waters Corporation, Milford, MA, USA), the peak detection and alignment were performed. The data were all subsequently normalized to the total ion intensity of each chromatogram, and the data matrices obtained were introduced into the EZinfo 2.0 software (Waters Corporation, Milford, MA, USA) for principal component analysis (PCA) and OPLS-DA. The metabolite peaks were assigned based on an MS^E^ analysis or were inferred using available biochemical databases such as the HMDB (http://www.hmdb.ca/), ChemSpider (http://www.chemspider.com/) and KEGG (http://www.kegg.com/). Potential markers were identified from the constructed S plots after the OPLS-DA, and the markers were selected based on their involvement in the variation and correlation of the dataset.

The generated sequences were first filtered, and then the reads that contained numerous interspersed Ns in their sequences or comparatively short reads (<17 bp) were not included in subsequent evaluations. The sequence reads remaining following the filtering quality control were subsequently analysed using the CLC Genomics Workbench 4. After mapping, the unique gene reads for all of the 17,108 annotated chicken genes in the database from the six libraries were combined and analysed using the DESeq R package^52^. The DEGs between the susceptible and resistant groups were identified at combined cut-offs with P < 0.05 and a fold-change >2. Functional annotations for the DEGs and the statistical analysis of the significantly represented functional categories were performed using DAVID^53–55^. Significance was set at a fold-enrichment >2, P < 0.05, and a false discovery rate (FDR) <20% for the pathway analysis.

Other statistical analyses were carried out using the statistical package for the social science (SPSS) 17.0 (SPSS Inc., Chicago, IL. USA). Differences between groups were tested using the *t*-test for independent samples. Significant differences were defined as P < 0.05 and the data were presented as the means ± standard deviation (SD).

## References

[1] Baghbanzadeh A, Decuypere E (2008). Ascites syndrome in broilers: physiological and nutritional perspectives. Avian Pathol..

[2] Balog JM (2003). Ascites syndrome (pulmonary hypertension syndrome) in broiler chickens: are we seeing the light at the end of the tunnel?. Avian Poult. Biol. Rev..

[3] Currie RJ (1999). Ascites in poultry: recent investigations. Avian Pathol..

[4] Wideman RF, Rhoads DD, Erf GF, Anthony NB (2013). Pulmonary arterial hypertension (ascites syndrome) in broilers: a review. Poultry Sci..

[5] Nicholson JK, Lindon JC, Holmes E (1999). ‘Metabonomics’: understanding the metabolic responses of living systems to pathophysiological stimuli via multivariate statistical analysis of biological NMR spectroscopic data. Xenobiotica.

[6] Hendriksen PJ (2007). Transcriptomics analysis of interactive effects of benzene, trichloroethylene and methyl mercury within binary and ternary mixtures on the liver and kidney following subchronic exposure in the rat. Toxicol. Appl. Pharmacol..

[7] Yum S, Woo S, Kagami Y, Park HS, Ryu JC (2010). Changes in gene expression profile of medaka with acute toxicity of Arochlor 1260, a polychlorinated biphenyl mixture. Comp. Biochem. Physiol. C Toxicol. Pharmacol..

[8] Griffin JL (2004). An integrated reverse functional genomic and metabolic approach to understanding orotic acid-induced fatty liver. Physiol. Genomics.

[9] Borgan E (2010). Merging transcriptomics and metabolomics–advances in breast cancer profiling. BMC Cancer.

[10] Zhang Y, Zhang X, Wu B, Cheng S (2012). Evaluating the transcriptomic and metabolic profile of mice exposed to source drinking water. Environ. Sci. Technol..

[11] Wu B (2012). Responses of mouse liver to dechlorane plus exposure by integrative transcriptomic and metabonomic studies. Environ. Sci. Technol..

[12] Li H (2008). Transcriptomic and metabonomic profiling of obesity-prone and obesity-resistant rats under high fat diet. J. Proteome Res..

[13] Zhou M (2012). Transcriptomic and metabonomic profiling reveal synergistic effects of quercetin and resveratrol supplementation in high fat diet fed mice. J. Proteome Res..

[14] Hoving-Bolink AH, Kranen RW, Klont RE, Gerritsen CL, de Greef KH (2000). Fibre area and capillary supply in broiler breast muscle in relation to productivity and ascites. Meat Sci..

[15] Julian RJ (1993). Ascites in poultry. Avian Pathol..

[16] Moghadam HK, Mcmillan I, Chambers JR, Julian RJ, Tranchant CC (2005). Heritability of sudden death syndrome and its associated correlations to ascites and body weight in broilers. Brit. Poultry Sci..

[17] Aksit M, Altan O, Karul A, Balkaya M, Ozdemir D (2008). Effects of cold temperature and vitamin E supplementation on oxidative stress, Troponin-T level, and other ascites-related traits in broilers. Arch. Geflugelk..

[18] Ozkan S (2007). Dietary vitamin E (alpha-tocopherol acetate) and selenium supplementation from different sources: performance, ascites-related variables and antioxidant status in broilers reared at low and optimum temperatures. Brit. Poultry Sci..

[19] Burton RR, Besch EL, Smith AH (1968). Effect of chronic hypoxia on the pulmonary arterial blood pressure of the chicken. Amer. J. Physiol..

[20] Hakim TS (1988). Erythrocyte deformability and segmental pulmonary vascular resistance: osmolarity and heat treatment. J. Appl. Physiol..

[21] Mirsalimi SM, Julian RJ (1993). Effect of excess sodium bicarbonate on the blood volume and erythrocyte deformability of broiler chickens. Avian Pathol..

[22] Yang Y, Gao M, Wu Z, Guo Y (2010). Genistein attenuates low temperature induced pulmonary hypertension in broiler chicks by modulating endothelial function. Eur. J. Pharmacol..

[23] Mandegar M (2004). Cellular and molecular mechanisms of pulmonary vascular remodeling: role in the development of pulmonary hypertension. Microvascular Res..

[24] Nong Z, Stassen JM, Moons L, Collen D, Janssens S (1996). Inhibition of tissue angiotensin-converting enzyme with quinapril reduces hypoxic pulmonary hypertension and pulmonary vascular remodeling. Circulation.

[25] Hanshaw RG, Smith BD (2005). New reagents for phosphatidylserine recognition and detection of apoptosis. Bioorgan. Med. Chem..

[26] Van Meer G, Simons K, Op DKJ, Van Deenen LM (1981). Phospholipid asymmetry in Semliki Forest virus grown on baby hamster kidney (BHK-21) cells. Biochemistry.

[27] Daleke DL, Lyles JV (2000). Identification and purification of aminophospholipid flippases. Biochim. Biophys. Acta.

[28] Kidd PMP (1996). Membrane Nutrient for Memory. A Clinical and Mechanistic Assessment. Altern. Med. Rev..

[29] Calderon C, Huang ZH, Gage DA, Sotomayor EM, Lopez DM (1994). Isolation of a nitric oxide inhibitor from mammary tumor cells and its characterization as phosphatidyl serine. J. Exp. Med..

[30] Martin SJ (1995). Early redistribution of plasma membrane phosphatidylserine is a general feature of apoptosis regardless of the initiating stimulus: inhibition by overexpression of Bcl-2 and Abl. J. Exp. Med..

[31] Fadeel B (1999). Phosphatidylserine exposure during apoptosis is a cell-type-specific event and does not correlate with plasma membrane phospholipid scramblase expression. Biochem. Biophys. Res. Commun..

[32] Pelassy C, Breittmayer JP, Aussel C (2000). Regulation of phosphatidylserine exposure at the cell surface by the serine–base exchange enzyme system during CD95-induced apoptosis. Biochem. Pharmacol..

[33] Fadok VA, de Cathelineau A, Daleke DL, Henson PM, Bratton DL (2001). Loss of phospholipid asymmetry and surface exposure of phosphatidylserine is required for phagocytosis of apoptotic cells by macrophages and fibroblasts. J. Biol. Chem..

[34] Ackerstaff E, Glunde K, Bhujwalla ZM (2003). Choline phospholipid metabolism: a target in cancer cells?. J. Cell. Biochem..

[35] Glunde K, Serkova NJ (2006). Therapeutic targets and biomarkers identified in cancer choline phospholipid metabolism. Pharmacogenomics.

[36] Heine W, Radke M, Wutzke KD (1995). The significance of tryptophan in human nutrition. Amino Acids.

[37] Feng B (2007). Metabolic profiling analysis of a D-galactosamine/lipopolysaccharide-induced mouse model of fulminant hepatic failure. J. Proteome Res..

[38] Ishimori K (1992). Site-directed mutagenesis in hemoglobin: functional and structural role of inter- and intrasubunit hydrogen bonds as studied with 37 beta and 145 beta mutations. Biochemistry.

[39] Dodgson JB, Mccune KC, Rusling DJ, Krust A, Engel JD (1981). Adult chicken alpha-globin genes alpha A and alpha D: no anemic shock alpha-globin exists in domestic chickens. Proc. Nat. Acad. Sci. USA.

[40] Steck TL (1974). The organization of proteins in the human red blood cell membrane. A review. J. Cell Biol..

[41] Huang CH, Liu PZ, Cheng JG (2000). Molecular biology and genetics of the Rh blood group system. Semin. Hematol..

[42] Van Kim CL, Colin Y, Cartron JP (2006). Rh proteins: key structural and functional components of the red cell membrane. Blood Rev..

[43] Soupene E (2002). Rhesus expression in a green alga is regulated by CO(2). Proc. Nat. Acad. Sci. USA.

[44] Endeward V (2006). Evidence that aquaporin 1 is a major pathway for CO2 transport across the human erythrocyte membrane. Faseb J..

[45] Geng AL, Guo YM, Yang Y (2004). Reduction of ascites mortality in broilers by coenzyme Q10. Poultry Sci..

[46] NRC. Nutrient Requirements of Poultry. 9th ed. (National Academies Press, 1994).

[47] Wang Y (2012). Changes of hepatic biochemical parameters and proteomics in broilers with cold-induced ascites. J. Anim. Sci. Biotechno..

[48] Julian RJ, Frazier JA, Goryo M (1989). Right ventricular hypertrophy, right ventricular failure and ascites in broiler chickens caused by Amiodarone-induced lung pathology. Avian Pathol..

[49] Herget J (1991). Pathophysiology of the pulmonary blood vessels in chronic lung disease. Physiol Res..

[50] Barth PJ, Kimpel C, Roy S (1993). An improved mathematical approach for the assessment of the medial thickness of pulmonary arteries. Pathol. Res. Pract..

[51] Tan X (2005). Activation of PKCalpha and pulmonary vascular remodelling in broilers. Res. Vet. Sci..

[52] Anders S, Huber W (2010). Differential expression analysis for sequence count data. Genome Biol..

[53] Dennis GJ (2003). DAVID: Database for Annotation, Visualization, and Integrated Discovery. Genome Biol..

[54] Huang D, Chang TR, Aggarwal A, Lee RC, Ehrlich HP (1993). Mechanisms and dynamics of mechanical strengthening in ligament-equivalent fibroblast-populated collagen matrices. Ann. Biomed. Eng..

[55] Huang DW, Sherman BT, Lempicki RA (2009). Systematic and integrative analysis of large gene lists using DAVID bioinformatics resources. Nat. Protoc..

[56] Druyan S (2007). The expression patterns of hypoxia-inducing factor subunit alpha-1, heme oxygenase, hypoxia upregulated protein 1, and cardiac troponin T during development of the chicken heart. Poultry Sci..

